# In Situ Control of the Eluted Ni Nanoparticles from Highly Doped Perovskite for Effective Methane Dry Reforming

**DOI:** 10.3390/nano12193325

**Published:** 2022-09-24

**Authors:** Heesu Kim, Rasika Mane, Kyeongwon Han, Hyungjin Kim, Chanmin Lee, Yukwon Jeon

**Affiliations:** 1 Department of Environmental and Energy Engineering, Yonsei University, 1 Yonseidae-gil, Wonju 26493, Korea; 2 Green and Sustainable Materials R&D Department, Korea Institute of Industrial Technology, 89 Yangdaegiro-gil, Ipjan-myeon, Seobuk-gu, Cheonan-si 31056, Korea

**Keywords:** Ni, perovskite, exsolution, size control, methane dry reforming

## Abstract

To design metal nanoparticles (NPs) on a perovskite surface, the exsolution method has been extensively used for efficient catalytic reactions. However, there are still the challenges of finding a combination and optimization for the NPs’ control. Thus, we report in situ control of the exsolved Ni NPs from perovskite to apply as a catalyst for dry reforming of methane (DRM). The La_0.8_Ce_0.1_Ti_0.6_Ni_0.4_O_3_ (LCTN) is designed by Ce doping to incorporate high amounts of Ni in the perovskite lattice and also facilitate the exsolution phenomenon. By control of the eluted Ni NPs through exsolution, the morphological properties of exsolved Ni NPs are observed to have a size range of 10~49 nm, while the reduction temperatures are changed. At the same time, the chemical structure of the eluted Ni NPs is also changed by an increased reduction temperature to a highly metallic Ni phase with an increased oxygen vacancy at the perovskite oxide surface. The optimized composite nanomaterial displays outstanding catalytic performance of 85.5% CH_4_ conversion to produce H_2_ with a value of 15.5 × 10^11^ mol/s·gcat at 60.2% CO conversion, which shows the importance of the control of the exsolution mechanism for catalytic applications.

## 1. Introduction

In recent years, perovskite oxides have been investigated for various means of green energy production due to their thermal stability and intrinsic catalytic activity [1]. In general, perovskite oxides have a formula structure of ABO_3_, consisting of a cation A site, a cation B site, and oxygen. Their various properties, such as their catalytic properties, can be effectively controlled according to the targeted intention [2,3], since various materials can be freely doped to the B site from transition metals (e.g, Ni, Co, and Mn) [4,5,6] to precious metals (e.g, Pd, Rh, and Ru) [7,8,9].

Currently, in the development of efficient catalysts, researchers have suggested to carefully design effective metal nanoparticles (NPs) on a support surface [10]. Nevertheless, these NPs are often a challenge, mainly as a result of agglomeration or coarsening, which causes a significant loss in catalytic activity. To overcome these problems, the “inside-out” method known as the exsolution technique has been intensively studied [11]. In site-deficient perovskite structures, B-site metals can be eluted as NPs from the surface in a reducing atmosphere [12,13,14,15,16,17,18,19,20]. It is widely demonstrated that the eluted NPs are basically embedded in the support surface in the form of sockets, and they do not easily aggregate even at high temperatures. When there are fewer A-site cations than B-site cations (A/B < 1), a perovskite oxide lattice with an excess portion of B-site cations is formed. A relatively large number of transition metal cations at the B-site can be easily eluted in a reducing atmosphere because A-site-deficient perovskites have a more stable form during exsolution than the form in which the ratio of A-site cations to B-site cations is 1:1 [11,12,13]. Nowadays, it has been reported that eluted NPs can be controlled by temperature reductions and time for different numbers, sizes, relationships with support, and structural changes of NPs [13,14].

Perovskite oxide nanomaterials with a strong chemical bonding structure have been widely used as catalysts for dry reforming of methane (DRM), owing to its high resistance to carbon deposition in a hydrocarbon atmosphere [13,17,18,19,20]. The DRM reaction is an important process because it can produce energetic syngas of H_2_ and CO from methane and carbon dioxide, which are greenhouse gases [19]. DRM requires a high temperature (e.g., 900–1273 K) and highly endothermic reaction due to the stability of the C-H bond and C=O:

CH_4_ + CO_2_ = 2CO + 2H_2_ △H°_298_ = 247 kJ/mol (1)

Hence, noble and non-noble metal-based catalysts are widely used for DRM reactions. Although noble metals (e.g., Rh, Ru, Pd, and Pt) can display excellent catalytic activity and stability with carbon deposition resistance, their application at the industrial level remains a big challenge due to the high cost [10,21]. Therefore, Ni-based catalysts have been suggested as a catalyst for DRM, having high reactivity and a relatively low cost. 

However, applying transition metals to the perovskite structure may show an unstable structure, where the element is not doped due to the size or large amount of doping. This has been shown in Ni-doped perovskite, where the Ni was not completely doped into the structure due to the large doping level inside the perovskite structure protruding out of the arrangements [22,23,24]. To solve this drawback, previous studies have suggested an augmented space for Ni doping through doping on relatively bigger Ce (IV) (Å = 0.87) [24] in the perovskite lattice, resulting in an ion radius increase for doped Ni species by a large amount (i.e., over 13 wt%) [25,26]. However, Ce generally contains an unstable orbital shell which has a large change in the intermediate valence depending on the environment, so it is thermodynamically unstable [27]. This is one reason why major studies have been conducted on the exsolution of Ni NPs on Sr-doped perovskites in changing reduction temperatures [28]. Therefore, although Ce-doped perovskites can be a good candidate for support due to their great catalytic properties in oxygen mobility, they require more studies to understand the exsolution mechanism and control the NPs’ shape, size, and distribution on the perovskite surface for effective usage in various applications.

In this paper, we present an eluted Ni NP shaping process and the changes in size, distribution, and structure on the Ce-doped perovskite oxide surface. With a large amount of Ni doping, the Ni NPs on the surface during exsolution were controlled through the change in the firing temperature in a reducing atmosphere. Moreover, the relationship between the A and B sites in the perovskite oxide with the strongly bonded Ni NPs is investigated. To verify the shape effect of the exsolved NPs and the synergy effects with the Ce-doped perovskite oxide surface structure, we compared the catalytic performances at the DRM reactions.

## 2. Materials and Methods

### 2.1. Synthesis of Ni Exsolved Perovskite Oxides

Lanthanum (III) oxide (La_2_O_3_, >99.9%), cerium (IV) oxide (CeO_2_, >99.9%), titanium (IV) oxide (TiO_2_, anatase, >99.9%), and nickel (II) oxide (NiO, >99.9%) were purchased from Sigma-Aldrich Co., Ltd. (St. Louis, MO, USA). La_0.8_Ce_0.1_Ti_0.6_Ni_0.4_O_3_ (LCTN) was prepared by solid state synthesis [11]. Non-stoichiometric amounts of the precursors were mixed with acetone and a small amount of a non-aqueous dispersant (Hypermer KD-1). Then, the acetone was evaporated at room temperature under stirring. The obtained powder was calcined at 1000 °C for 12 h to decompose the carbonates. To form the highly crystalline perovskite phase, the calcined powder was pressed into pellets and calcined at 1400 °C for 12 h. Subsequently, to exsolve the Ni NPs on the surface, the calcined La_0.8_Ce_0.1_Ti_0.6_Ni_0.4_O_3_ (Ni = 13.2 wt%) was treated at different temperatures of 600 °C, 700 °C, and 800 °C at a rate of 5 °C/min under a continuous gas flow of 5% H_2_/N_2_ for 12 h.

### 2.2. Characterization

The X-ray diffraction (XRD) patterns of the samples were measured with a D2 phaser (Bruker AXS) using a Ni-filtered Cu-Kα X-ray source. The generator operated at 30 kV and 10 mA. Scans were performed within the 2θ range of 20–80, and the step size was 0.02/s. The NP size and distribution were observed by field emission scanning electron microscope (FE-SEM) images using a JEOL-7800F (JEOL). To analyze the NP morphology, transmission electron microscope (TEM) and energy dispersive spectroscopy (EDS) mapping analysis were performed using a JEM-ARM 200F NEOARM (JEOL). The chemical bonding structures were investigated using X-ray photoelectron spectroscopy (XPS) (Thermo Fisher, Waltham, MA, USA) with an Al X-ray source (1486.7 eV). All the binding energies were referenced to the C1 peak at 284.8 eV.

### 2.3. Dry Reforming of Methane (DRM)

The catalytic activity was assessed in a fixed-bed quartz tube (9.5-mm inside diameter) reactor, where 200 mg of sample powder was placed in the middle of the reactor and reduced in situ in a 5% H_2_/N_2_ atmosphere at 800 °C for 2 h. The gas mixture of CH_4_, CO_2_, and N_2_ was used with each flow rate of 15, 15, and 90 mL min^−^^1^, respectively, for a dry reforming of methane reaction with a gas volumetric flow rate (GHSV) of 36,000 mL g_cat_**^−^**^1^ h^−^^1^. The gaseous products were analyzed online by gas chromatography (Agilent GC 8890) with a dual system of a thermal conductivity detector (TCD) and a flame ionization detector (FID) using a Porapak Q column and a Molecular Sieve 5A packed column, respectively. To evaluate the performance, the conversion (%) and reactivity (mol/s ∙ g_cat_) for CH_4_ and CO_2_ were calculated according to the following equations:

CH_4_ Conversion (%) = [CH_4_]_consumed_/[CH_4_]_feed_ × 100%

CO_2_ Conversion (%) = [CO_2_]_consumed_/[CO_2_]_feed_ × 100%

CH_4_ Reactivity (mol/s ∙ g_cat_) = [H_2_]_detect_**/**(2 × weight of catalyst)

CO_2_ Reactivity (mol/s ∙ g_cat_) = [CO]_detect_**/**(2 × weight of catalyst)

## 3. Results and Discussion

### 3.1. Synthesis and Characterization of Ni-Eluted LCTN

**Figure 1:**
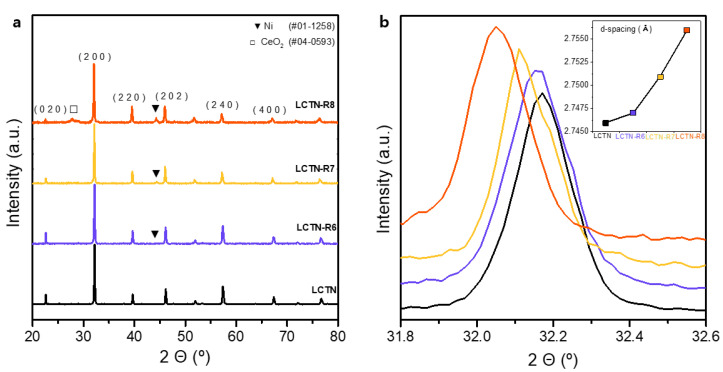
XRD pattern images of LCTN, LCTN-R6, LCTN-R7, and LCTN-R8 at (**a**) full range and (**b**) magnified range for (200) peak (inset: change in d-values).

We synthesized the highly Ni-doped perovskite oxide La_0.8_Ce_0.1_Ti_0.6_Ni_0.4_O_3_ (LCTN) with the design of an A site-deficient structure using Ce species under solid state synthesis conditions. Figure 1 presents the X-ray diffraction (XRD) patterns for the LCTN before and after the reduction process. We found that the XRD pattern of the original LCTN basically adopted an orthorhombic Pbnm structure. A highly crystalline and pure single phase without any impurity peaks was achieved, although over 13 wt% of the Ni species was doped in the perovskite due to the increased ionic space from the large Ce ion in the oxide lattice.

To elute the Ni NPs on the surface and control the NP size, a reduction process under various conditions was also conducted at temperatures of 600, 700, and 800 °C, and these samples are denoted as LCTN-R6, LCTN-R7, and LCTN-R8, respectively. Overall, the XRD patterns showed no change in the original perovskite with an orthorhombic Pbnm structure. However, as the reduction temperature increased, the main reflections gradually shifted toward lower 2θ values. This can be more obviously seen in Figure 1b, which displays the magnified XRD peaks for the detailed crystalline phase characteristics in the (200) plane and its d-value changes. The calculated lattice parameters of the d-value and cell volume are summarized in Table 1. The angle of the (200) reflection decreased with an increasing reduction temperature, while the d-value increased from 2.746 Å to 2.756 Å and the cell volume enlarged from 242.18 Å^3^ to 245.15 Å^3^, which is ascribed to the lattice expansion originated by the formation of oxygen vacancies upon reduction and possibly Ni exsolution [29].

As can be seen in Figure 1a, the Ni exsolutions for LCTN-R6, LCTN-R7, and LCTN-R8 were also confirmed by the Ni metal peaks (JCPDS # 01-1258) to appear at around 44.37°. Interestingly, these also shifted to a lower 2θ (Table 1), which was related to the unit cell volume expansion [15]. The doped Ni^2+^ (ionic radius = 0.69 Å) [24] was reduced to Ni^0^ (atomic radius = 1.63 Å), resulting in unit cell expansion. This strongly indicates that the Ni NPs were successfully exsolved on the surface, which will be discussed further in relation to the subsequent XPS analysis. The relatively bigger change in the unit cell expension also supports that more Ni species may be taken out to the surface. This can be expected from the increased Ni NP size at higher exsolution temperatures caluated in Table 1, showing the possiblity of the eluted Ni NPs’ size control.

**Figure 2:**
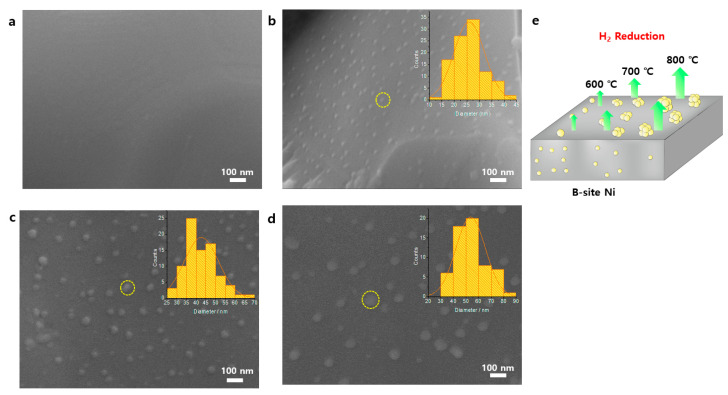
SEM images (inset: particle size distribution) of (**a**) LCTN, (**b**) LCTN-R6, (**c**) LCTN-R7, (**d**) LCTN-R8, and (**e**) the expected exsolution mechanism.

To confirm and compare the exsolution of Ni NPs, morphological analysis by scanning electron microscopy (SEM) images with the NP size distributions was performed (Figure 2) for the pristine LCTN, LCTN-R6, LCTN-R7, and LCTN-R8. For the non-treated LCTN (Figure 2a), a dense and smooth surface was observed, indicating a single phase even in the presence of a large amount of Ni on the B-site, which was previously confirmed by the XRD analysis (Figure 1). On the other hand, LCTN-R6, LCTN-R7, and LCTN-R8 clearly showed eluted Ni NPs on their perovskite surfaces in comparison with that of the pristine LCTN. Generally, the exsolution of metallic NPs occurs via destabilization of the parent perovskite lattice with high deficiencies in the A site, which are reported to facilitate a change in the equilibrium position, driving exsolution, and oxygen positions [13]. The fine exsolution process in our perovskite system may have been due to this reason, where the doped relatively bigger Ce provided not only an ionic space for high Ni doping but also a high deficiency to facilitate Ni exsolution to the surface.

As calculated from Figure 2b–d, the average NP sizes of LCTN-R6, LCTN-R7, and LCTN-R8 were also changed by approximately 26, 43, and 54 nm, respectively, agreeing with a similar trend for the XRD results in Table 1. As illustrated in the expected exsolution mechanism (Figure 2e), it can clearly be seen that a higher reduction temperature tended to nucleate more Ni NPs and also increase the size of the particles from the carefully designed A-site deficient perovskite structure.

**Figure 3:**
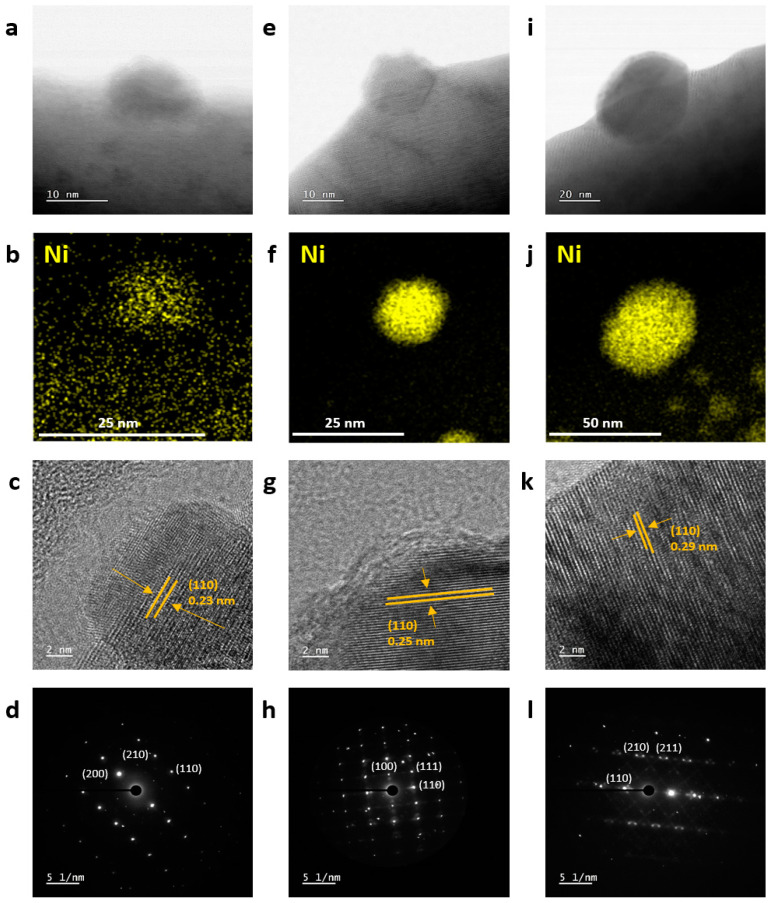
Morphological characterization of Ni-exsolved perovskite oxides, showing HR-TEM images, EDS mapping, and SAED patterns of (**a**–**d**) LCNT-R6, (**e**–**h**) LCNT-R7, and (**i**–**l**) LCNT-R8.

To further confirm the crystallographical structure and eluted Ni NPs, high-resolution transmission electron microscopy (HR-TEM) and energy dispersive X-ray spectroscopy (EDS) analyses were performed for the NPs at LCTN-R6, LCTN-R7, and LCTN-R8. As shown in Figure 3a,e,i, the eluted NPs were embedded into the perovskite surface, which is understandable in the exsolution process due to the NP synthesis from the inside structure. This feature might help the synergy effect with oxide support by strong interaction with the enhanced catalytic properties and also provide resistance to NP agglomeration during the DRM reaction. The embedded NPs showed different sizes, with a diameter of approximately 14~49 nm on the LCTN surface, which is in perfect agreement with the previous SEM images.

Aside from that, the EDS results in Figure 3b,f,j show that the Ni atoms only existed within the eluted NPs. It is implied that thermal treatment under reducing conditions results in the formation of NPs, which can be identified to be enriched by Ni at the surface. Notably, Ni species remained at the bulk LCTN-R6 pervoskite, while most of the Ni species were taken out to the surface for LCTN-R7 and LCTN-R8. This implies that the exsolution may start from the reduction temperature of 600 °C and transform the large amount of Ni species to all metals, which is important to increase and control the actual active sites for the DRM reaction.

Interestingly, as shown in Figure 3c,g,k, the lattice spacing values of LCTN-R6, LCTN-R7 and LCTN-R8 increased (0.23, 0.25, and 0.29 nm, respectively), indicating the (110) plane of the Ni lattice. With a similar metallic Ni cubic structure with the [002], [001] and [001] zone axis confirmed by the selected area electron diffraction (SAED) patterns in Figure 3d,h,l, the strain in the Ni NPs could also be changed due to the metallic Ni amount exsolving to the perovskite surface, in close relation with the catalytic activity in any kind of reaction.

**Figure 4:**
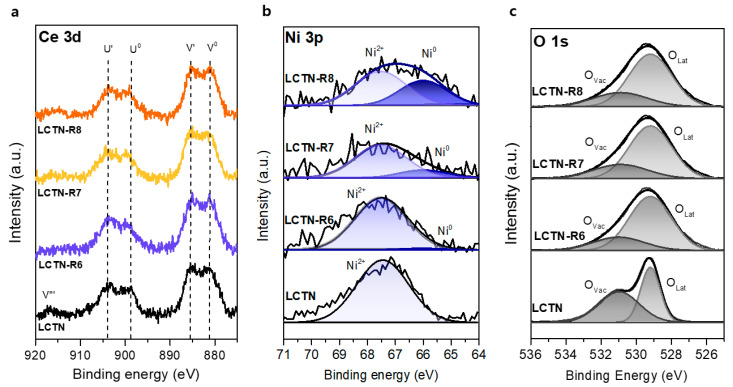
XPS spectra of LCTN, LCTN-R6, LCTN-R7, and LCTN-R8: (**a**) Ni 3p, (**b**) O 1s, and (**c**) Ce 3d.

X-ray photoelectron spectroscopy (XPS) analysis was conducted to elucidate the changes in the chemical bonding structure caused by the reduction treatment. Figure 4 displays the XPS spectrum of LCTN before and after reduction, corresponding to Ce 3d, Ni 3p, and O 1s. The oxidation states of each element were confirmed by XPS measurements except La, because overlap of the La 3d_3/2_ peak with the main Ni 2p peak may have led to low accuracy of the oxidation state estimation [15,30]. As shown in Figure 4a, The Ce 3d XPS spectra were split into Ce^3+^ and Ce^4+^. Two pairs of peaks at approximately 899, 881.6, 903.2, and 884.9 eV were characteristic of Ce in the 3+ oxidation state, corresponding to Ce 3d 4f^2^L (U^0^ and V^0^) and Ce 3d 4f^1^L (U’ and V’), respectively [31]. The observed peak at a binding energy of 916.6 eV, which was clearly found in LSTN, corresponded to Ce 3d 4f^0^L (V’’’) in the 4+ oxidation state. When changing the reduction temperatures, the peak shape and position were similar. The slight decrease in the area of the 916.6-eV peak after reduction indicates that Ce was predominantly in the 3+ oxidation state, which explains how Ce stably maintained doping to the A site in a reducing atmosphere.

Figure 4b shows the Ni 3p region, with the proportion ratios summarized in Table 2. The peaks that appear at 66.0 eV and 67.5 eV are the typical 3p binding energies of Ni^0^ and Ni^2+^, respectively. As the reduction temperature increased from 600 to 700 and 800 °C, the Ni^0^ fraction increased from 5.06% to 21.46% and 30.09%, respectively. It is likely that more Ni species were reduced to metal by increasing the reduction temperature. Additionally, the Ni metal in the LCTN bulk lattice continuously exsolved to the surface as Ni NPs under the reducing environment, which was correlated with the TEM analysis (Figure 3).

In general, oxygen deficiencies in metal oxides lead to the formation of oxygen vacancies (V_O_), which can significantly affect a material’s behavior, such as its catalytic activity [32]. Regarding the O species in Table 2, which were calculated from Figure 4c, the oxygen vacancies were 46.0% before the reduction treatment, which was probably due to the A-site defect. It is worth noting that an A-site defect of 0.1 mol results in non-stoichiometry of the surface oxygen and facilitates exsolution of the doped B-site cation. After reduction treatment, the relative number of surface oxygen vacancies changed significantly (Table 2), which was related to the increased oxygen deficiency [33]. The oxygen vacancy was formed inside the support, owing to the protrusion of Ni species from the Ni-O bond inside the bulk [29,34]. Therefore, we can say that the oxygen vacancies increased in support when the exsolution of Ni increased for an enhanced catalytic property.

### 3.2. Catalytic Perperties for the DRM Reaction

**Figure 5:**
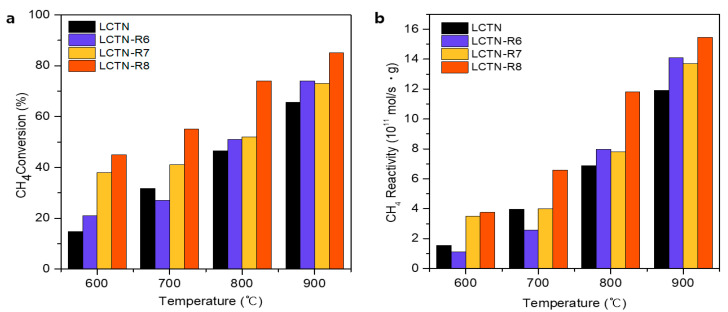
(**a**) CH_4_ conversion and (**b**) CH_4_ reactivity for H_2_ production rate.

**Figure 6:**
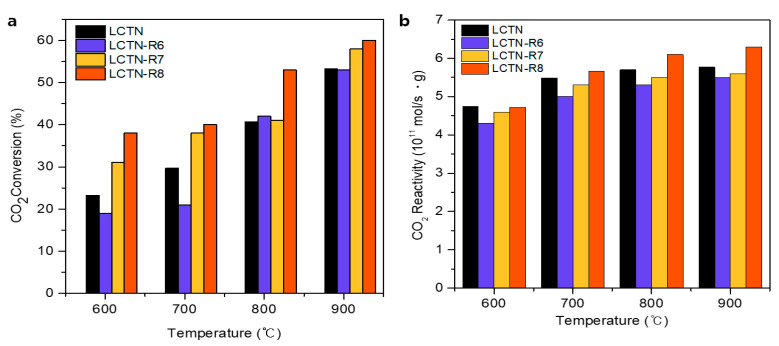
(**a**) CO_2_ conversion and (**b**) CO_2_ reactivity for CO production rate.

The catalytic activities of the prepared LCNT, LCTN-R6, LCTN-R7, and LCTN-R8 were investigated for the dry reforming of methane reaction at temperatures from 600 to 900 °C with a reaction time of 2 h and 1:1 molar ratio of CH_4_ to CO_2_. Figure 5 and Figure 6 show the CH_4_ and CO_2_ conversions with the CH_4_ and CO_2_ reactivities for the H_2_ and CO production rates, respectively. Overall, the reduced LCTN-R6, LCTN-R7, and LCTN-R8 catalysts showed improved CH_4_ reactivity compared with the pristine LCTN without a reduction treatment, which explains how the exsolved Ni NPs could enhance the H_2_ production rate for the DRM due to formation of the sufficient metal active sites. In particular, the LCTN-R8 showed a noticeable improvement in CH_4_ conversion of 85.5% with a high H_2_ production rate of 15.5 × 10^11^ mol/s·gcat, as shown in Figure 5. This was because the amount of Ni metallic species eluted to the surface increased as the reduction temperature increased, even though it had a relatively larger NP size, as confirmed by analysis on the morphology (see Figure 1 and Figure 3) and the chemical structure (see Figure 4 and Table 2).

As indicated by Figure 6a, it was possible to investigate the effect of the oxygen vacancy on the DRM reaction through the CO_2_ conversion of pristine LCTN, which was greater than those of LCTN-R6 (with relatively less metallic Ni-active sites) at 600 and 700 °C and also similar at higher reaction temperatures. These results implied that the relatively higher amount of oxygen vacancy presented in the pristine LCTN perovskite support could enhance the CO_2_ adsorption and dissociation. Moreover, the CO_2_ reactivities (Figure 6b) of LCTN-R6 and LCTN-R7 were found to be lower than that of pristine LCTN, which was probably due to their reduced oxygen vacancy in comparison with LCTN, resulting from the XPS of O ls (Figure 4 and Table 2).

**Figure 7:**
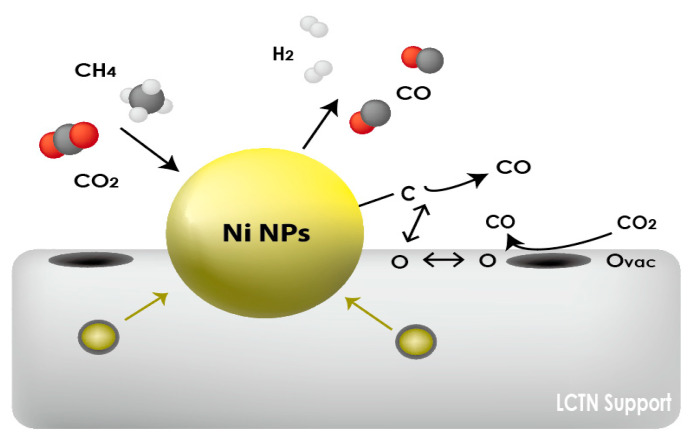
Schematic diagram of reaction mechanism of DRM reaction over eluted Ni NPs on the perovskite catalysts.

As indicated in Figure 5 and Figure 6, the overall CO_2_ conversion and CH_4_ reactivity of LCTN-R8, with values of 60.2% and 6.3 × 10^11^ mol/s·gcat, respectively, were higher than those of other catalysts due to the sufficient NP size with mainly metallic Ni species and more oxygen vacancies. Meanwhile, the higher CO production rate can also mean less carbon deposition on the Ni NPs, since it is easily oxidized to CO due to the facilated oxygen ion transport to the surrounding Ni NPs [35]. In summation, Figure 7 proposed the schematic diagram of the reaction mechanism of the DRM reaction over eluted Ni NPs on the perovskite catalysts. Therefore, we can conclude that the highly exposed metallic Ni NPs on the surface with a higher oxygen vacancy can be effective for dry reforming of methane processes.

## 4. Conclusions

In this work, we investigated the in situ control of the eluted Ni NPs from highly doped La_0.8_Ce_0.1_Ti_0.6_Ni_0.4_O_3_ perovskite oxides using the solid state method followed by reduction treatment. An A-site deficient perovskite with high Ni loading was achieved by doping relatively bigger Ce ions to the lattice structure, which also facilitated the Ni exsolution. To control the exsolved Ni NPs, the reduction temperatures were changed from 600 to 800 °C. From the results of SEM, TEM, and XPS analysis, the temperature increase promoted the synthesis of eluted Ni NPs with a highly metallic phase during the reduction process. The formation of surface oxygen was accelerated since the doped Ni species moved to the surface and made oxygen vacancies, which affected the catalytic activity. These synthesized composite nanomaterials with eluted Ni NPs on the surface were utilized as catalysts for the dry reforming of methane reaction. The LCTN perovskite reduced at 800 °C exhibited the highest catalytic performance for dry reforming of methane, which was concluded to be due to the formation of the highly metallic Ni-active sites and relatively higher oxygen vacancy with a sufficient size for the exsolved Ni NPs. Therefore, we are proposing an optimization strategy of exsolution and recommend a promising catalyst structure for the dry reforming of methane.

## Figures and Tables

**Table 1:** Crystalline characterization from XRD results.

Catalysts	(200) Peak (°)	*d* (Å)	Cell Volume (Å^3^)	Ni Metal Peak (°)	Ni Metal Crystallite Size ^1^ (Å)
LCTN	32.17	2.746	242.18	-	-
LCTN-R6	32.15	2.748	243.04	44.385	27.163
LCTN-R7	32.11	2.751	244.42	44.364	30.752
LCTN-R8	32.05	2.756	245.15	44.283	34.295

^1^ NP size was calculated from the Scherrer formula based on the XRD measurement.

**Table 2:** Binding energies and peak areas for Ni 2p and O 1s XPS spectra.

Catalysts	Ni 2p		O 1s	
	BE (eV) ^1^	Area (%) ^2^	BE (eV) ^1^	Area (%) ^2^
LCTN	67.45 (Ni^2+^)	100	529.2 (O_Lat_) 531 (O_Def_)	46.14 53.86
LCTN-R6	67.5 (Ni^2+^) 66 (Ni^0^)	94.94 5.06	529.2 (O_Lat_) 531 (O_Def_)	77.10 22.90
LCTN-R7	67.5 (Ni^2+^) 66 (Ni^0^)	78.54 21.46	529.2 (O_Lat_) 530.9 (O_Def_)	74.44 25.56
LCTN-R8	67.5 (Ni^2+^) 66 (Ni^0^)	69.91 30.09	529.15 (O_Lat_) 531 (O_Def_)	56.53 43.47

^1^ Measurement binding energy (eV) and ^2^ relative percentages (%).

## Data Availability

Not applicable.
